# New Prehospital Triage for Stroke Patients Significantly Reduces Transport Time of EVT Patients Without Delaying IVT

**DOI:** 10.3389/fneur.2021.676126

**Published:** 2021-06-11

**Authors:** Martin Cabal, Linda Machova, Daniel Vaclavik, Petr Jasso, David Holes, Ondrej Volny, Michal Bar

**Affiliations:** ^1^Department of Neurology - Comprehensive Stroke Centre, University Hospital Ostrava, Ostrava, Czechia; ^2^First Faculty of Medicine, Charles University in Prague, Prague, Czechia; ^3^Faculty of Medicine, Masaryk University, Brno, Czechia; ^4^Agel Research and Training Institute, Vitkovice Hospital, Ostrava, Czechia; ^5^Faculty of Medicine, Ostrava University, Ostrava, Czechia; ^6^Emergency Medical Service of Moravian-Silesian Region, Ostrava, Czechia; ^7^Jessenius Faculty of Medicine, Commenius University in Bratislava, Martin, Slovakia; ^8^Czech National Centre for Evidence-Based Healthcare and Knowledge Translation (Cochrane Czech Republic, Czech EBHC: JBI Centre of Excellence, Masaryk University GRADE Centre), Institute of Biostatistics and Analyses, Faculty of Medicine, Masaryk University, Brno, Czechia

**Keywords:** prehospital triage, stroke, paramedic, EVT, large vessel occlusion

## Abstract

**Background and Purpose:** Ischemic stroke is a leading cause of mortality and morbidity worldwide. The time from stroke onset to treatment impacts clinical outcome. Here, we examined whether changing a triage model from “drip and ship” to “mothership” yielded significant reductions of onset-to-groin time (OGT) in patients receiving EVT and onset-to-needle time (ONT) in IVT-treated patients, compared to before FAST-PLUS test implementation. We also investigated whether the new triage improved clinical outcomes.

**Methods:** In a before/after multicenter study, we evaluated the effects of changing the prehospital triage system for suspected stroke patients in the Moravian–Silesian region, Czech Republic. In the new system, the validated FAST PLUS test is used to differentiate patients with suspected large vessel occlusion and triage-positive patients are transported directly to the CSC. Time metrics and patient data were obtained from the regional EMS database and SITS database.

**Results:** For EVT patients, the median OGT was 213 min in 2015 and 142 min in 2018, and the median TT was 142 min in 2015 and 47 min in 2018. For tPA patients, the median ONT was 110 min in 2015 and 109 min in 2018, and the median TT was 41 min in 2015 and 48 min in 2018. Clinical outcome did not significantly change. The percentages of patients with favorable clinical outcome (mRS 0–2) were comparable between 2015 and 2018: 60 vs. 59% in tPA patients and 40 vs. 44% in EVT patients.

**Conclusions:** The new prehospital triage has yielded shorter OGTs for EVT patients. No changes were found in the onset-to-needle time for IVT-treated patients, or in the clinical outcome at 3 months after stroke onset.

## Introduction

Ischemic stroke is a leading cause of mortality and morbidity worldwide (1), and the recognition of stroke symptoms and prehospital stroke management represent critical bottlenecks in acute stroke management. The delay from the onset of stroke symptoms to hospital arrival is largely due to delayed activation by the patient/witness or failure of EMS crew to recognize stroke symptoms (2–4). Delayed hospital arrivals are common and contribute to the fact that only one in four stroke patients present within the time window for receiving tPA (5). Quicker therapy provision is associated with better clinical outcome (6–8); therefore, effective prehospital intervention is important.

**Figure 1:**
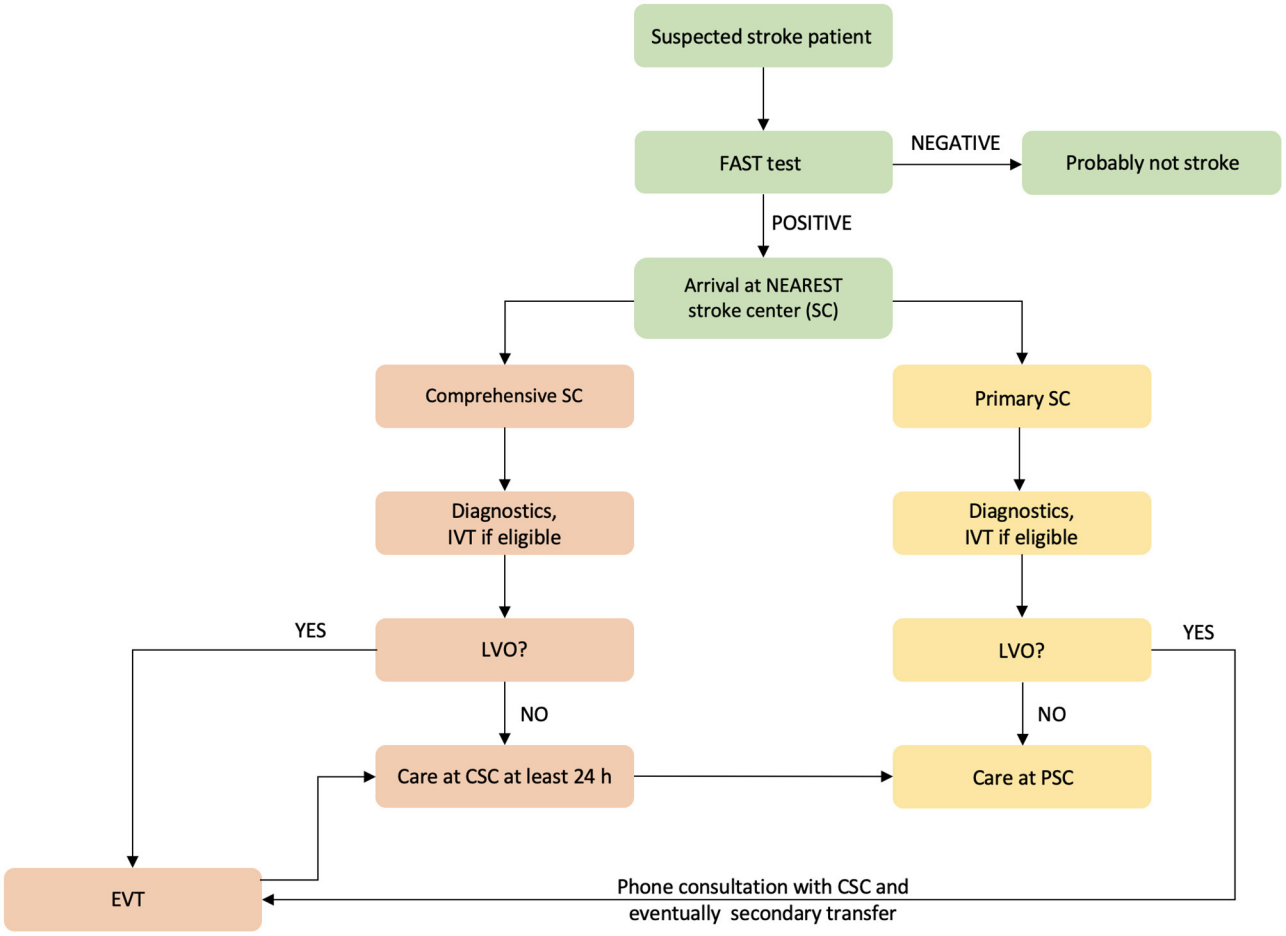
Flowchart of prehospital triage in the Moravian–Silesian region, Czech Republic, before the triage change. IVT indicates intravenous thrombolysis; EVT, endovascular treatment; and LVO, large vessel occlusion.

**Figure 2:**
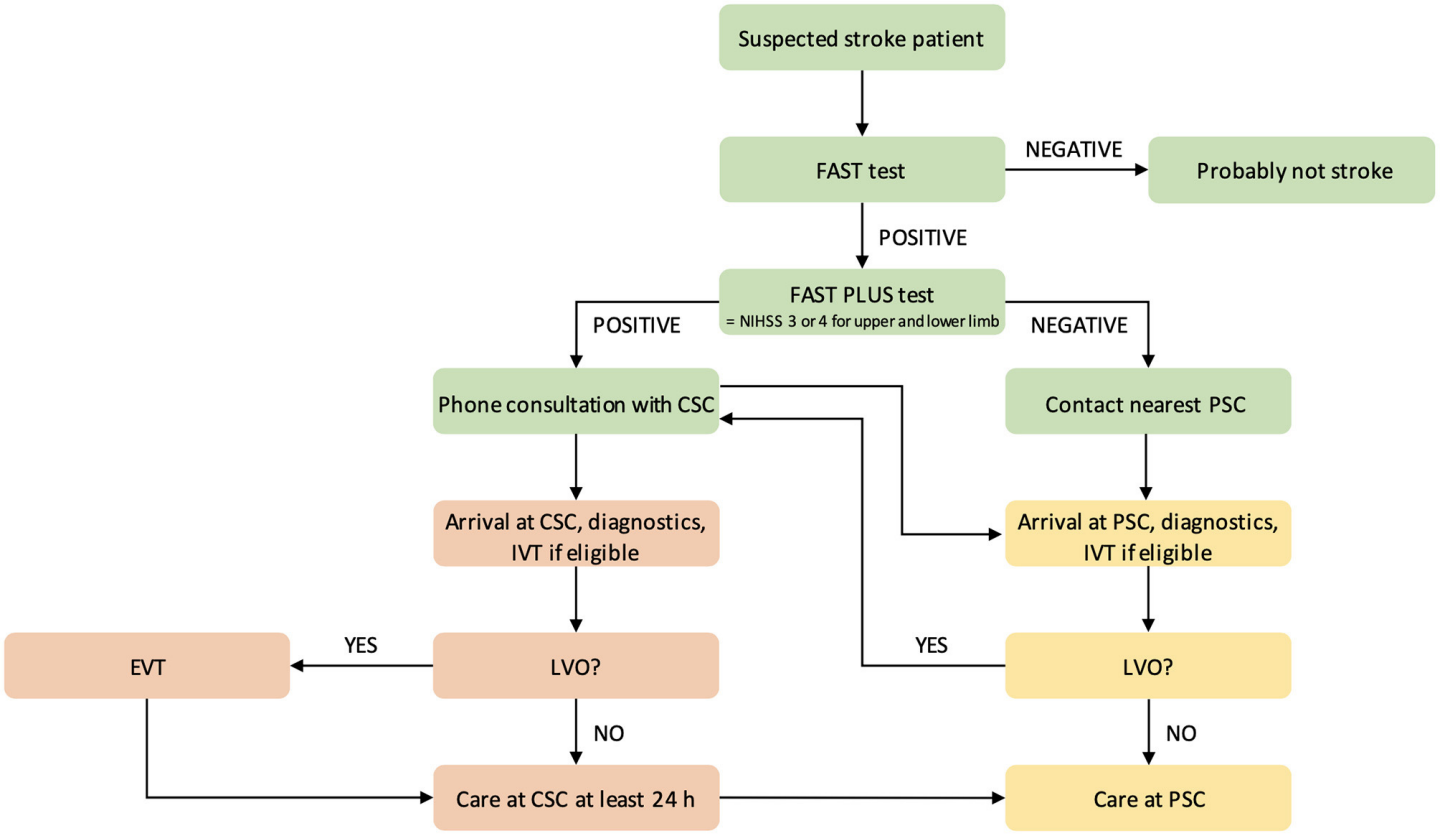
Flowchart of prehospital triage in the Moravian–Silesian region, Czech Republic, after the triage change. CSC indicates comprehensive stroke center; PSC, primary stroke center; IVT, intravenous thrombolysis; EVT, endovascular treatment; and LVO, large vessel occlusion.

One possible means of decreasing prehospital delay is to increase stroke preparedness in the general population; however, previous studies of this topic have shown mixed results (9–11). Another method is to educate paramedics in stroke signs recognition. This method was used in the Czech Republic before the implementation of a new prehospital triage test (12). In 2016, a prehospital stroke scale, the FAST PLUS test, which evaluates for the presence of severe hemiparesis (NIHSS of three to four points for upper and lower limbs), was introduced in the Moravian–Silesian region to differentiate patients with suspected large vessel occlusion (LVO). This test reportedly predicts LVO with 93% sensitivity and 47% specificity (13). If the FAST PLUS result is positive, the paramedic has a tele-consultation with a hospital-based neurologist in a comprehensive stroke center (CSC). If the neurologist agrees, the triage-positive patient is transferred directly to the CSC (mothership approach). If the FAST PLUS test result is negative, the nearest primary stroke center is contacted. With the introduction of the FAST PLUS test, the triage model has been changed from drip and ship (transferring the stroke patient to the nearest stroke center, and if there is LVO, then secondary transfer to CSC is indicated) (Figure 1) to mothership (direct transfer of stroke patient with suspected LVO to CSC—i.e., bypassing the nearest PSC) (Figure 2).

Currently, there is only a limited body of evidence comparing these prehospital models (mothership vs. drip and ship) (14, 15).

The main aim of our present study was to determine whether changing the triage model from drip and ship to mothership yielded a significant reduction in onset-to-groin time (OGT) among patients receiving EVT and in onset-to-needle time (ONT) among IVT patients, compared to the situation before FAST-PLUS test implementation. The second aim was to determine whether the new triage protocol led to better clinical outcomes.

## Methods

In this study, we conducted a before/after study comparing the situation before and after a change in prehospital stroke triage system. In 2016, a new prehospital triage test (FAST PLUS test) was introduced to detect stroke patients with possible LVO in the Moravian–Silesian region of the Czech Republic. Before its implementation in routine clinical practice, this test was validated and demonstrated the following: sensitivity 93%, specificity 47%, PPV 41%, and NPV 94% (13). Its inter-rater reliability was assessed and showed moderate agreement between paramedics and neurologists (12).

### Population

**Figure 3:**
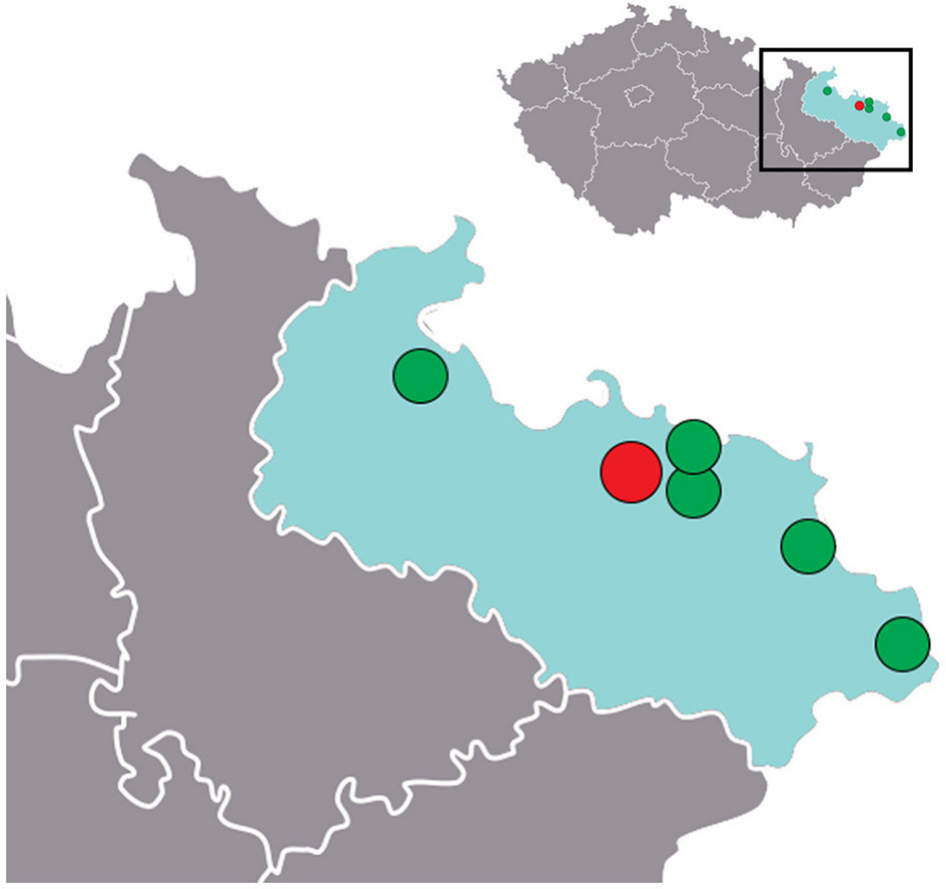
Stroke centers in the Moravian–Silesian region of the Czech Republic (gray color). Green circles, PSC; red circle, CSC.

The Moravian–Silesian region is home to ~1.2 million inhabitants, with a population density of 220/km^2^. It includes five primary stroke centers (PSCs) and one CSC, with a maximum driving distance of ~50 km to the nearest stroke center and ~95 km to the nearest CSC. Figure 3 presents the locations of the PSCs and CSC in this region. In 2015 and 2018, one PSC performed mechanical thrombectomies only for patients from its primary catchment area (300,000 inhabitants).

### Emergency and Triage System

There is only one regional Emergency Medical Services (EMS) system, and the majority of stroke patients are transported by ground EMS transport.

Under the mothership model, patients with positive FAST PLUS results are transported directly to the CSC. The transport times of all stroke patients treated with IVT, EVT, or both at all stroke centers in the Moravian–Silesian region were compared between 2015 (before the intervention) and 2018 (after). From the regional EMS database, we got the list of patients transferred with suspected stroke to all stroke centers in the region and all the time metrics of these transfers.

### Data Collection

**Figure 4:**
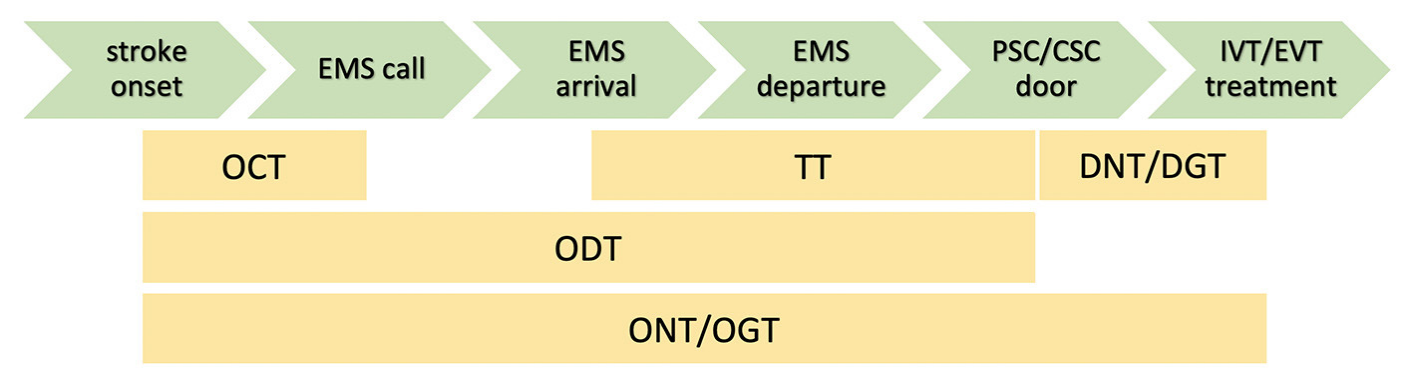
Calculated time metrics. OCT indicates onset-to-call time; TT, transfer time; DNT, door-to needle time; DGT, door-to-groin time; ODT, onset-to-door time; ONT, onset-to-needle time; and OGT, onset-to-needle/groin time.

Using the Safe Implementation of Treatment in Stroke (SITS) database, we obtained the time metrics of therapeutic interventions, patient identification, age, sex, baseline NIHSS, and clinical outcome evaluated by modified Rankin scale (mRS) at 3 months after stroke onset, door-to-needle time (DNT) or door-to-groin time (DGT), and stroke onset time. The onset time was completed for every patient, using either the stroke onset time or last seen well time from the SITS database. We calculated the following time metrics (Figure 4): onset-to-call time (OCT, time from the first stroke symptoms to EMS call by patient or witness), transfer time (TT, time from the arrival of paramedics at the stroke site to arrival at hospital), onset-to-door time (ODT, from stroke onset to door of hospital), onset-to-needle time (ONT, ODT + time to the first bolus of IVT), and onset-to-groin time (OGT, ODT + time to groin puncture).

The SITS initiative offers a platform for collecting stroke data from stroke centers in more than 80 countries. The registry is internet based, which allows rapid data entry and retrieval, and allows centers to compare their own treatment results on both a national and global scale.

To achieve the data completeness, an official e-mail has been sent to the chairs and physicians of all included stroke centers, who were responsible for data entering into the SITS database. The centers were officially asked to update all relevant time metrics as well as outcome measures (3-month modified Ranking scale). The centers were not aware of the study neither before it was started or after the study was finished.

All data are available on reasonable request from the corresponding author.

### Statistical Analysis

Data were processed using standard statistical analysis methods and reported as median values, means, standard deviations, contingency intervals, and IQR variance. Testing was performed using Kruskal–Wallis-type non-parametric tests. Normality was assessed by Shapiro–Wilk test. Analyses were performed using the “R-project” package of mathematical libraries.

This study was approved by the Ethical Committee of University Hospital Ostrava, Czech Republic, and is registered at ClinicalTrials.gov (identifier: NCT03072524). For this type of study, informed consent is not required.

## Results

In 2015, a total of 3,513 patients were diagnosed with acute ischemic stroke, of whom 431 were treated either with tPA or mechanical thrombectomy (EVT)—including 364 (85%) with tPA only and 89 (20%) with EVT ± tPA. In 2018, a total of 3,554 patients were diagnosed with acute ischemic stroke, of whom 691 were treated−654 (95%) with tPA only and 179 (26%) with EVT ± tPA. Between these time periods, the number of patients treated with endovascular intervention doubled from 89 to 179. The 2015 and 2018 populations did not significantly differ in sex distribution: 54% men in 2015 vs. 52% men in 2018 (*P* = 0.5). The median age was 72 years in 2015 and 74 years in 2018 (Table 1).

**Table 1:** Demographic data and results.

	**2015**		**2018**		***P*****-value**	
	**IVT**	**EVT**	**IVT**	**EVT**	**IVT**	**EVT**
Total number of treated patients	431		691		NA	
Number of patients, total (% men)	364 (54.4)	89 (56.2)	654 (52)	179 (54.7)	NS	NS
Age	72.5 (16.2)	70 (12)	74 (16)	73 (12)	0.170	0.024
Baseline NIHSS	11 (9.5)	16 (9)	9 (11)	16 (7)	0.003	0.307
Favorable clinical outcome (mRS 0–2), %	60	40	59	44	NA	NA
DNT, min	45 (24)	—	26 (15)	—	<0.001	NA
DGT, min	—	32,5 (74.2)	—	60 (35)	NA	0.023
OCT, min	15 (54.1)	9 (11)	12 (28.3)	7 (17.6)	0.024	0.515
TT, min	41 (19.7)	142 (128.1)	48 (20.1)	47 (19.7)	<0.001	<0.001
ODT, min	93 (111)	179 (184.8)	81 (44.4)	74 (34.9)	<0.001	<0.001
ONT, min	110 (81)	—	109 (48.7)	—	0.118	NA
OGT, min	—	213 (105)	—	142 (51.5)	NA	<0.001

*Data presented as median (IQR) unless otherwise indicated*.

*DNT indicates door-to-needle time; DGT, door-to-groin time; OCT, stroke onset to call of the EMS; TT, transfer time (from the arrival of EMS to a stroke patient to the stroke center); ODT, stroke onset to arrival to the stroke center; ONT, onset-to-needle time (only for IVT patients); and OGT, onset to the catheterization of femoral artery (only for EVT patients)*.

Among tPA-treated patients, the median TT was 41 min (IQR 19.7) in 2015 and 48 min (IQR 20.1) in 2018 (*P* < 0.001), and the median ONT was 110 min (IQR 81) in 2015 and 109 min (IQR 48.7) in 2018. Among EVT-treated patients, the median TT was 142 min (IQR 128.1) in 2015 and 47 min (IQR 19.7) in 2018 (*P* < 0.001), and the median OGT was 213 min (IQR 105) in 2015 and 142 min (IQR 51.5) in 2018 (*P* < 0.001). Among all stroke patients, the median DNT was 45 min (IQR 24) in 2015 and 26 min (IQR 15) in 2018 (*P* < 0.001). The median DGT increased from 33 min (IQR 74.2) in 2015 to 60 min (IQR 35) in 2018 (*P* = 0.02).

In 2015, from 53 secondary transferred patients, 49 received EVT (92.4%). In year 2018, 14 out of 21 received EVT (66.7%).

**Figure 5:**
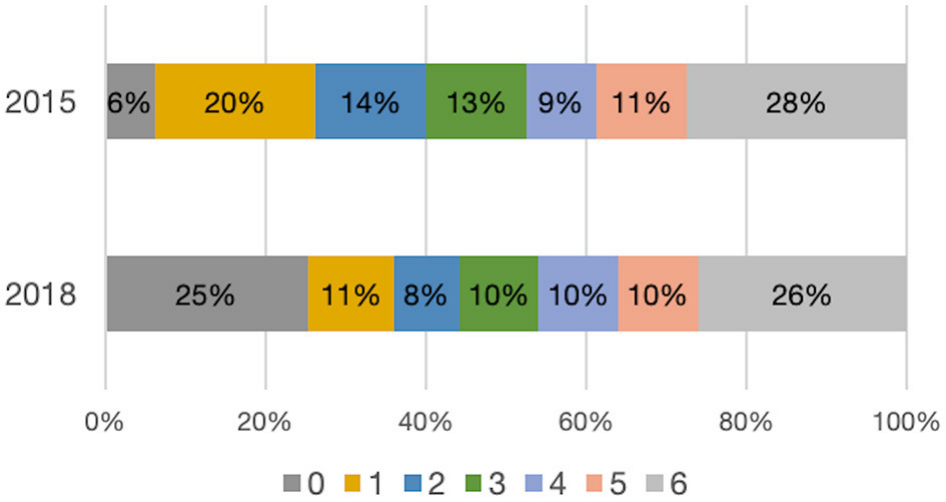
Distribution of mRS values at 3 months for EVT patients in 2015 and 2018.

The percentages of patients with favorable clinical outcome (mRS 0–2) were comparable between 2015 and 2018: 60 vs. 59% among tPA-treated patients and 40% vs. 44% among EVT-treated patients (Figure 5).

## Discussion

Prior to implementation of the new prehospital triage test, it was uncertain whether the bypass of PSC might delay IVT initiation. Among EVT patients, the median TT was three times shorter in 2018 compared to in 2015 (*P* < 0.001). On the other hand, among tPA patients, the median TT was 7 min longer in 2018 (48 vs. 41 min) because triage-positive patients were not taken to the nearest PSC but rather directly to the CSC, which was sometimes farther. Nevertheless, the median ONT (onset-to-needle time) for tPA patients was 110 in both 2015 and 2018 due to the shortening of intrahospital times (median DNT decreased from 45 to 26 min). Moreover, we observed a significant positive change in OGT from 213 min in 2015 to 142 min in 2018 (*P* < 0.001).

Notably, the number of endovascular interventions practically doubled between 2015 and 2018. There are probably multiple reasons for this, including better availability of this therapeutic intervention, increasing clinical experience, and changes of the guidelines for patient selection. The number of secondary transfers decreased, but the median DGT increased from 33 to 60 min (*P* = 0.02). This increase was possibly due to the need to perform diagnostic tests at the CSC before endovascular intervention.

In our opinion, the lower median baseline NIHSS in 2018 might be partially explained by the effect of national strategy to support the IVT administration in acute stroke patients. A very recent publication by Mikulik et al. (16) focused on this topic and demonstrated that it is feasible and achievable to treat as many as 20% of all ischemic stroke patients with IVT. Continuous education of neurologists and less fear of IVT administration in low/er NIHSS patients might be another possible explanation for this phenomenon.

Our secondary aim was to determine whether the change of triage method has led to better clinical outcomes. The study by Herm et al. (17) focused on analysis of the DGT within the German Stroke Registry of EVT-treated patients and its impact on functional outcome. Fifty-six percent of the patients were primarily treated in CSC and 44% of the patients were primarily treated in PSC and then transferred to CSC. Median DGT was shorter in the PSC-treated patients (44 min); in CSCs, the median DGT was 79 min. On the other hand, median OGT was 196 min among patients primarily transferred to CSC vs. 278 min for patients primarily treated in PSC. In our study, we have shown very similar findings. The median DGT was 33 min in 2015 and 60 min in 2018 (mothership strategy). The median OGT shortened from 213 min in 2015 to 142 min in 2018. One possible explanation of prolonged DGT, when more patients were primarily treated in CSC, is that the lower number of secondary transports (with diagnostic CT and CT angiography performed in the PSCs) led to a higher number that need to perform diagnostic tests at CSC before EVT. Nevertheless, the percentage of EVT patients with a favorable clinical outcome (mRS 0–2) did not change overtime (44 in 2018 vs. 40% in 2015).

The percentages of patients with favorable clinical outcome treated with IVT (i.e., mRS 0–2 at 3 months) did not change either (60% in 2015 and 59% in 2018).

When comparing the transport situation in the Moravian–Silesian region with the mathematical models presented in the paper by Schlemm et al. (18), there is a similarity with the rural scenario (rectangular shape with one side 120 km and the second side 60 km). Schlemm et al. concluded that if the additional delay to IVT is <50 min, the patient with suspected LVO should be transferred directly to CSC. In the Moravian–Silesian region, the longest on-road distance between PSC and CSC is 50 km (for road-based EMS transport, it is <50 min); therefore, our effort to use the mothership model for suspected LVO patients corresponds to the study results of Schlemm et al.

The triage change was also accompanied by the lower number of secondary transfers of stroke patients with LVO between PSC and CSC from 53 in 2015 to 21 in 2018. In 2015, from 53 secondary transferred patients, 49 received EVT (92.4%). In 2018, 14 out of 21 received EVT (66.7%). The main reasons for not performing the EVT in 2018 were complete recanalization on the first DSA run (three patients) and operator/technical difficulties of reaching the occlusion (four patients).

A similar study was recently conducted in Stockholm, with a comparison of the situations before and after implementation of the SSTS (Stockholm Stroke Triage System) for predicting LVO (19). Their study was also region-specific, was conducted over 1 year (October 2017–October 2018), and included patients transported to the hospital for suspected acute stroke. Their primary objective was to evaluate the performance of the SSTS, which is highly similar to the FAST PLUS test. Both tests evaluate upper and lower limb weakness. The only difference is the NIHSS cutoff value, which is ≥3 for each limb with the FAST PLUS, and ≥2 with the SSTS. Implementation of the SSTS yielded the same results as found in our present study: shortening of onset-to-puncture/groin time without delaying IVT. The authors mentioned several study limitations, such as the specific region and logistic circumstances, the large number of patients without emergent CT angiography scans, and not reporting comparisons of clinical outcomes.

The presently observed shortening of OGT is similar to results reported in other PSC bypass studies using the RACE (Rapid Arterial oCclusion Evaluation) scale (20, 21) or LAMS (Los Angeles Motor Scale) score (22). However, these previous studies did not analyze all of the time metrics assessed in our study (e.g., ONT, ODT, and TT).

## Study Limitations

This study was conducted in a specific geographic locality with relatively short distances between the PSCs and CSC. We are aware that our data were based on a retrospective analysis of registry data (SITS) and their quality depends on accuracy and completeness.

In addition, the secular trends must be taken into the consideration—change of lifestyle, improved public awareness, or increased lifespan.

## Conclusions

The change in prehospital triage yielded shortening of the OGT among EVT patients, as well as a reduction of the number of secondary transfers from 53 to 21. No changes were observed in the onset-to-needle time among IVT-treated patients or in clinical outcome at 3 months after stroke onset. Mothership triage is supported by the results of our study in case of stroke patients with severe hemiparesis.

## Data Availability Statement

The data analyzed in this study was obtained from the Safe Implementation of Treatment in Stroke (SITS) database with the cooperation of those responsible for granting access to this data. Requests to access these datasets should be directed to SITS International, info@sitsinternational.org.

## Author Contributions

MC, LM, DV, OV, and MB contributed to conception and design of the study. DH and PJ organized the cooperation with paramedics. MC organized the database and cooperated with statistician. MC and OV wrote the first draft and all revised versions of the manuscript. All authors contributed to manuscript revision, read, and approved the submitted version.

## Conflict of Interest

The authors declare that the research was conducted in the absence of any commercial or financial relationships that could be construed as a potential conflict of interest.

## Abbreviations

CSC: comprehensive stroke center
DGT: door-to-groin time
DNT: door-to-needle time
EMS: emergency medical services
EVT: endovascular treatment
IVT: intravenous thrombolysis
LVO: large vessel occlusion
OCT: onset-to-call time
ODT: onset-to-door time
OGT: onset-to-groin time
ONT: onset-to-needle time
PSC: primary stroke center
SSTS: stockholm stroke triage system
tPA: tissue plasminogen activator
TT: transport time.
